# Folate‐Associated Gene Expression in Primary Tumors Is Associated With Tumor Response and Progression‐Free Survival of Patients With Metastatic Colorectal Cancer Undergoing 5‐FU/Leucovorin‐Based Combination Chemotherapy

**DOI:** 10.1002/cam4.70895

**Published:** 2025-05-13

**Authors:** Elisabeth Odin, Göran Carlsson, Pushpa Saksena, Anders Edsjö, Alessandro Di Cara, Roger Tell, Bengt Gustavsson, Yvonne Wettergren

**Affiliations:** ^1^ Department of Surgery, Institute of Clinical Sciences Sahlgrenska Academy, University of Gothenburg Gothenburg Sweden; ^2^ Department of Surgery Sahlgrenska University Hospital, Region Västra Götaland Gothenburg Sweden; ^3^ Department of Pathology Sahlgrenska University Hospital Gothenburg Sweden; ^4^ Department of Clinical Genetics, Pathology, and Molecular Diagnostics Office for Medical Services, Region Skåne Lund Sweden; ^5^ Division of Pathology, Department of Clinical Sciences Lund University Lund Sweden; ^6^ Lonza, Biologics R&D Basel Switzerland; ^7^ Isofol Medical AB Gothenburg Sweden

## Abstract

**Background:**

5‐Fluorouracil (5‐FU) and the Folate Leucovorin (LV) form the chemotherapy backbone for metastatic colorectal cancer (mCRC). Tumoral expression of specific folate‐associated genes is associated with the risk of recurrence in stage III CRC following adjuvant 5‐FU/LV (FLV)‐based combination chemotherapy according to the Nordic bolus regimen. The aim was to evaluate whether expression of folate‐associated genes in Pre‐therapeutic tumor samples is associated with outcomes of patients with mCRC undergoing palliative FLV‐based combination chemotherapy.

**Patients and Methods:**

Patients treated with FLV (*n* = 113), FLV + oxaliplatin (FLOX, *n* = 102), or FLV + irinotecan (FLIRI, *n* = 75) were included. *ABCC3*, *RFC‐1, PCFT*, *MFT*, *MTHFD2*, and *TYMS* expression was determined by qPCR and related to tumor response and 3‐year progression‐free survival (PFS). Analyses were conducted on the entire cohort and on subgroups (group 1: stage I–III; group 2: stage IV, at primary surgery). Multivariate Cox proportional hazard models were applied to assess associations between covariates and PFS.

**Results:**

Low *TYMS* and high *MFT* expression in group 1, and high *ABCC3* expression in group 2 correlated with better PFS (HR 1.37 (1.04–1.82), HR 0.49 (0.30–0.80), and HR 0.74 (0.60–0.93)), respectively. In addition, high *MFT* expression was associated with better PFS of patients treated with FLIRI (HR 0.47 (0.27–0.81), *p* = 0.007) whereas high expression of *ABCC3* was associated with better PFS of patients treated with FLOX (HR 0.46 (0.23–0.92), *p* = 0.029).

**Conclusion:**

While pretherapeutic tumoral expression of specific folate‐associated genes may not serve as a universal predictive marker for FLV‐based treatment, it might predict response and outcomes in patients receiving FLOX or FLIRI. Evaluating the impact of gene expression in primary tumors should consider subgrouping of patients by disease stage at diagnosis as well as the applied chemotherapy regimen. Prospective clinical studies on patients with mCRC are warranted to validate these findings.

**Abbreviations**
5,10‐MeTHF: 5,10‐methylenetetrahydrofolate
5‐FU: 5‐fluorouracil
5‐MTHF: 5‐methyltetrahydrofolate
ABC: ATP‐binding cassette
ACTB: β‐actin
ADP: adenosine diphosphate
AIC: Akaike Information criteria
ANOVA: analysis of variance
ATP: adenosine triphosphate
CFTR: cystic fibrosis transmembrane conductance regulator
CI: confidence interval
CRC: colorectal cancer
DACH‐Pt: dichloro(1,2‐diaminocyclohexane)platinum(II)
DFS: disease‐free survival
DHF: dihydrofolate
DHFR: dihydrofolate reductase
dTMP: deoxythymidine monophosphate
dUMP: deoxyuridine monophosphate
ECOG: Eastern Cooperative Oncology Group
ER: endoplasmatic reticulum
FAD: flavin adenine dinucleotide
FADH_2_
: flavin adenine dinucleotide, reduced form
FdUMP: fluorodeoxyuridine monophosphate
FdUr: fluorodeoxyuridine
FFPE: formalin‐fixed paraffin‐embedded
FLIRI: 5‐fluorouracil + leucovorin + irinotecan
FLOX: 5‐fluorouracil + leucovorin + oxaliplatin
FLV: 5‐fluorouracil + leucovorin
FR: folate receptors
H+: proton
hCTR1: copper uptake transporter 1
HR: hazard ratio
LV: leucovorin
MFT: mitochondrial folate transporter
MRP: multidrug resistance protein
MTHFD1: methylenetetrahydrofolate dehydrogenase 1
MTHFD1L: methylenetetrahydrofolate dehydrogenase (NADP+ dependent) 1‐like
MTHFD2: methylenetetrahydrofolate dehydrogenase‐cyclohydrolase
MTHFR: methylenetetrahydrofolate reductase
MTHFS: methenyltetrahydrofolate synthetase
NADP: nicotinamide adenine dinucleotide phosphate
NADPH: nicotinamide adenine dinucleotide phosphate, reduced form
NS: not significant
OAT2: organic anion transporter 2
PAD: Pathological‐Anatomical Diagnosis
PCFT: proton‐coupled folate transporter
PFS: progression‐free survival
Pi: inorganic phosphate
PPIA: peptidylprolyl isomerase A
qPCR: quantitative polymerase chain reaction
RECIST: response evaluation criteria in solid tumors
RFC‐1: reduced folate carrier
SHMT1: serine hydroxymethyltransferase 1
SHMT2: serine hydroxymethyltransferase 2
SN‐38: active metabolite of irinotecan
THF: tetrahydrofolate
TK1: thymidine kinase 1
TOPO1: topoisomerase 1
TYMP: thymidine phosphorylase
TYMS: thymidylate synthase

## 1 Introduction

5‐Fluorouracil (5‐FU) has been a cornerstone in the chemotherapy regimen for metastatic colorectal cancer (mCRC) for over six decades [1]. Meta‐analyses indicate that biochemical modulation of 5‐FU with the folate leucovorin (LV) can significantly enhance objective treatment response rates in patients with mCRC, increasing from 11% to approximately 21% [2]. Subsequent enhancements in treatment efficacy were achieved in the early 2000s by combining 5‐FU/LV (FLV)‐based regimens with oxaliplatin or irinotecan, resulting in response rates soaring up to 50% in first‐line therapy [3, 4].

Currently, ongoing clinical trials are assessing novel treatment regimens involving targeted and immune‐modulating drugs, demonstrating promising outcomes across various cancer types. Unfortunately, their effectiveness remains limited to a small subset of CRC patients [5]. Despite the need to optimize treatments for patients receiving FLV, there's a paucity of studies dedicated to exploring the potential enhancement of LV or other folates' modulatory effects in these patients. Moreover, the absence of informative biomarkers has hindered the identification of patients unlikely to benefit from LV therapy, creating a significant knowledge gap that needs to be addressed.

**FIGURE 1:**
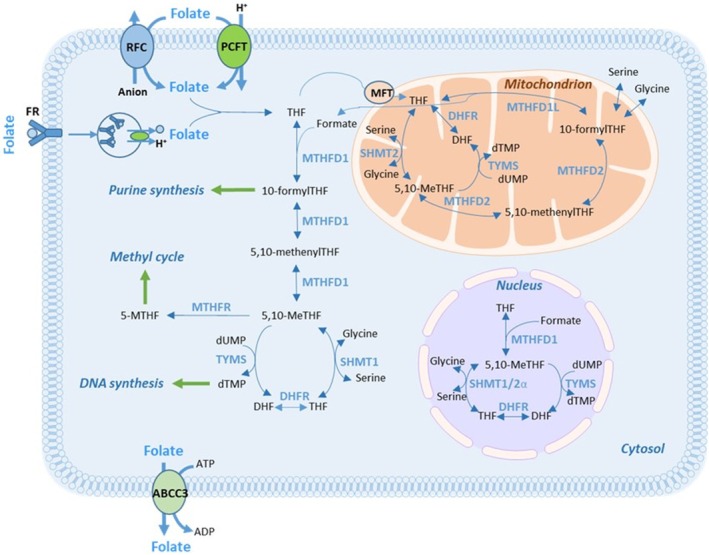
Simplified schematic representation of folate transport and metabolism in eukaryotic cells. Transport of folate into the cells may occur through the reduced folate carrier RFC‐1, the proton‐coupled folate transporter PCFT, or folate receptors (FR). MFT regulates folate metabolism via transport of THF into mitochondria. The enzymes MTHFD1 and SHMT1 are active in the cytosol, while MTHFD2, MTHFD1L, and SHMT2 are active in the mitochondrial compartment [6]. DHFR and TYMS are active in both the cytosol and the mitochondria. Sumoylated versions of DHFR, MTHFD1, SHMT1/2α, and TYMS are active in the nucleus [7]. MTHFR converts 5,10‐MeTHF to 5‐MTHF which enters the methyl cycle. Export of folates may occur via for example, ABCC3. Note that some of the reactions in the figure can be catalyzed by enzymes other than those depicted.

Low expression of folate‐associated genes can result in a poor response to FLV‐based therapy. This is because, without proper transportation and metabolism, LV cannot be converted to 5,10‐methylenetetrahydrofolate (5,10‐MeTHF), which serves as the co‐factor for the 5‐FU target enzyme, thymidylate synthase (TYMS). High levels of 5,10‐MeTHF are crucial for the formation of a stable ternary complex with TYMS and the 5‐FU metabolite FdUMP. This complex inhibits the generation of dTMP and subsequently hinders cell proliferation [1]. Some folate‐associated gene products participate in LV transportation into cells, whereas others are needed in intracellular folate polyglutamation or metabolism (Figure 1). The recent finding that the metabolic action of folates requires enzymes compartmentalized within the cytosol, the mitochondria, and the nucleus underscores the intricate nature of folate metabolism [7, 8].

It has been shown that administration of the standard dose of LV results in suboptimal tissue levels of 5,10‐MeTHF [9]. Furthermore, administration of 5,10‐MeTHF (arfolitixorin, formerly Modufolin) achieves higher levels of 5,10‐MeTHF in tumor tissue than both the racemic (LV) and natural (Isovorin) forms of 5‐formylTHF [10, 11]. Additionally, increasing doses of LV influence folate‐related gene expression, and there seems to be a correlation between the expression of the genes *ABCC3*, *PCFT*, and *RFC‐1* and folate concentrations in tumor tissue [12, 13]. Thus, it appears feasible to enhance the efficacy of FLV‐based chemotherapy by modulating TYMS inhibition using different forms and doses of folates, and possibly also to predict response to FLV‐based treatment using the expression level of folate‐associated genes.

Recently, we analyzed the expression of folate pathway genes in patients with stage III CRC treated with FLV alone, or FLV in combination with oxaliplatin (FLOX), according to the Nordic bolus regime [14, 15]. High expression of genes involved in folate transport, polyglutamation, or metabolism was significantly associated with decreased risk of recurrent disease. The informative genes either encoded mitochondrial enzymes or were regulated by mitochondrial transcription factors, highlighting the link to the mitochondrial one‐carbon pathway, which is consistently overexpressed in cancer [16]. Pretherapeutic expression of folate‐associated genes has also been reported to predict the success of neoadjuvant FLV‐based chemotherapy in patients with locally advanced adenocarcinomas of the esophagus [17].

The aim of the present study was to assess the association between primary tumor expression of six selected folate pathway genes, *ABCC3*, *RFC‐1*, *PCFT*, *MFT*, *MTHFD2*, and *TYMS*, and tumor response as well as 3‐year progression‐free survival (PFS) of patients with mCRC. The patients were subjected to palliative chemotherapy in the form of bolus FLV, FLOX, or FLIRI (i.e., FLV + irinotecan).

## 2 Patients and Methods

### 2.1 Study Design

A total of 290 patients with mCRC subjected to palliative treatment with FLV (*n* = 113), FLOX (*n* = 102) or FLIRI (*n* = 75) according to the Nordic bolus regime at the Sahlgrenska University Hospital during 1996–2012 were included in this retrospective study. The patients were followed for 3 years or until disease progression or death. All tumors were classified according to the Tumor–Node –Metastasis (TNM) staging system [18]. Gene expression levels were assessed in primary tumors and analyzed in relation to demographic, clinical, and pathological covariates including tumor response and 3‐year PFS. Analyses were conducted on the complete study cohort, as well as on patients stratified into two groups based on variations in the interval between primary surgery (i.e., tissue sampling) and the commencement of palliative chemotherapy. Group 1 comprised patients with stage I (*n* = 7), stage II (*n* = 51), and stage III (*n* = 84) disease who initiated palliative chemotherapy following relapse (with a median of 648 days after primary surgery, interquartile range 391–1099). Group 2 comprised patients diagnosed with stage IV disease (*n* = 148) at primary surgery who started palliative treatment as soon as possible after the surgical procedure (median 60 days, interquartile range: 43–85). Sixty‐one (72.6%) patients with stage III disease and four (7.8%) with stage II disease received adjuvant chemotherapy before palliative treatment. The duration from cessation of adjuvant therapy to the commencement of palliative treatment exceeded 6 months for 45 out of 65 patients.

### 2.2 Palliative Treatment

FLV treatment comprised intravenous bolus injection of 5‐FU (500 mg/m^2^), followed by bolus injection of LV (60 mg/m^2^) 30–40 min later, on Days 1 and 2 every other week, according to the Nordic FLV regime [19]. FLOX treatment comprised 5‐FU (500 mg/m^2^) and LV (60 mg/m^2^) bolus on Days 1 and 2 every other week, plus an oxaliplatin infusion (85 mg/m^2^) over 120 min on Day 1 every other week [20]. FLIRI treatment consisted of 5‐FU (500 mg/m^2^) and LV (60 mg/m^2^) bolus on Days 1 and 2 every other week, plus an irinotecan infusion (180 mg/m^2^) over 120 min on Day 1 every other week. Patients were assigned to the different chemotherapy regimens based on the following criteria: The standard first line chemotherapy for mCRC in the Nordic countries is FLIRI. However, elderly or frail patients were treated with the less intensive FLV regimen. FLOX treatment was reserved for patients who were considered for later surgical removal of liver metastases, as oxaliplatin causes less liver toxicity compared to irinotecan. The patients were followed for a maximum of 3 years or until the time of progression or death. Tumor response was evaluated by CT scans of the thorax/abdomen and characterized as complete response (CR), partial response (PR), stable disease (SD), or progressive disease (PR).

### 2.3 Histological Evaluation of Tissue Samples

Relevant formalin‐fixed paraffin‐embedded tissue (FFPE) blocks and their corresponding hematoxylin and eosin (H&E)‐stained slides were retrieved from the tissue archive. The selection process involved reviewing the technical aspects of the Pathological‐Anatomical Diagnosis (PAD) for each case. There was no information available about the specific tumor region from which the sample was taken, potentially introducing bias in the gene expression analysis due to intra‐tumoral heterogeneity. Only tumor tissue deriving from surgical specimens was used. After collecting all paraffin blocks and slides, the microscopic examination was done by an experienced anatomic pathologist with a special interest in gastrointestinal pathology. The tumor area with the highest percentage of tumor epithelial cells was identified on H&E‐stained slides. The corresponding area on the tissue block was then marked, microdissected, and transferred into RNase‐free tubes.

### 2.4 RNA Extraction, cDNA Preparation, and Quantitative Real‐Time PCR

A protocol for RNA extraction, cDNA preparation, and quantitative real‐time PCR (qPCR) was developed and optimized. Each part of the protocol was subsequently validated by an independent laboratory (TATAA Biocenter AB, Gothenburg, Sweden). Total RNA was extracted and purified using the AllPrep DNA/RNA FFPE kit (no. 80234, Qiagen) with modifications (Supporting Information—S1). cDNA was synthesized from total RNA using the SuperScript VILO Kit (Thermo Fisher Scientific) with modifications (Supporting Information—S1). The relative expression of the genes *ABCC3, RFC‐1, MFT, PCFT, MTHFD2*, and *TYMS* was determined using qPCR. The folate transporters *PCFT*, *RFC‐1*, and *ABCC3* were selected as they were the top three genes the expression of which was previously associated with disease‐free survival (DFS) in a study of patients with stage III CRC treated with adjuvant FLV [14]. *MFT* was included because it encodes a protein responsible for transporting folate into mitochondria. *MTHFD2* was added due to its role in converting 5,10‐methenylTHF to 5,10‐MeTHF within mitochondria. Finally, *TYMS* was included as it encodes an enzyme that forms a ternary complex with 5,10‐MeTHF in both the cytosol and mitochondria. The TaqMan Gene Expression Assays (Thermo Fisher Scientific) used are presented in Table S1. Each assay included a unique set of primers and a FAM‐MGB‐labeled probe that targeted a specific mRNA sequence. To prevent the amplification of genomic DNA, assays in which the probe spanned an exon–exon junction were selected. Further information regarding assay location in the genome, exon boundaries, and amplification length is available at https://www.thermofisher.com/se/en/home/life‐science/pcr/real‐time‐pcr/real‐time‐pcr‐assays/taqman‐gene‐expression/single‐tube‐taqman‐gene‐expression‐analysis.html.

The qPCR was set up in duplicates in 384‐well plates using a Nanodrop II (GC Biotech) and was carried out in 5 μL reactions as presented in Supporting Information—S1. The qPCR was run on a QuantStudio 12 K Flex Real‐Time PCR System (Thermo Fisher Scientific) according to a standard protocol. The thresholds and baselines were set manually in SDS, and Ct values were extracted. The variation between duplicates, calculated as the [(standard deviation/mean) × 100], was no more than 0.5% for any sample. Variations between runs were compensated for by normalization against a control sample. Delta Ct values were calculated by subtracting the mean Ct value of the reference genes β‐actin (ACTB) and peptidylprolyl isomerase A (PPIA) from the Ct value of the target gene. Thus, a high ΔCt value represents low gene expression and vice versa.

### 2.5 Statistical Analysis

Statistical analyses were performed using JMP 15.0.0 (SAS Institute, 2019), Graph Pad Prism version 9.3.1, 2021, R version 3.6.1, 2019, or IBM SPSS Statistics, version 28.0.1.0, 2021. Differences between groups were calculated using the Mann Whitney *U* test or the Wilcoxon/Kruskal‐Wallis' test, and data are presented as median and ranges. To compare sets of continuous parameters measured in the same sample, the Pearson correlation coefficient (*r*) was used. PFS was calculated from the start of chemotherapy treatment to the last follow‐up, or to the date of progression or death. Patients who were progression‐free at 3 years or who died during follow‐up were censored. A multi‐variable stepwise selection of variables using Akaike Information Criteria (AIC) was performed to identify a minimal set of variables associated with PFS. Cox proportional hazard regression was applied to assess potential associations between gene expression markers, clinical variables, and PFS. For all statistical analyses, *p* < 0.05 was considered significant.

## 3 Results

Patient and tumor characteristics, as well as the number of patients in relation to treatment regimens and tumor responses, are outlined in Table 1. As shown, there was no significant difference between groups 1 and 2 regarding sex, tumor location, or chemotherapy regimen; however, there were differences regarding age, ECOG performance status, and tumor differentiation. The median age of patients treated with FLV (72 years, range 38–84) was significantly higher (*p* < 0.0001) than the age of patients treated with FLOX or FLIRI (63 years, range 23–82).

**TABLE 1:** Patient and tumor characteristics, treatment regimens, and tumor response.

	Group 1, stage I–III^a^, (*n* = 142)	Group 2, stage IV^a^ (*n* = 148)	*p*	All patients (*n* = 290)
Sex, *n* (%)				
Female	59 (41.5)	75 (50.7)	0.12	134 (46.2)
Male	83 (58.4)	73 (49.3)		156 (53.8)
Median age, years (range)	69 (38–82)	64 (2384)	0.0013	66 (23–84)
ECOG performance status^b^, *n* (%)				
0	84 (59.2)	115 (77.7)	0.0007	199 (68.6)
1–2	58 (40.8)	33 (22.3)		91 (31.4)
Tumor differentiation, *n* (%)				
Well‐moderate	101 (71.1)	85 (57.4)	0.024	186 (64.2)
Poor	34 (23.9)	49 (33.1)		83 (28.6)
Mucinous	5 (3.5)	13 (8.8)		18 (6.2)
Not evaluated	2 (1.4)	1 (0.68)		3 (1.0)
Tumor location, *n* (%)				
Right‐sided colon	52 (36.6)	58 (39.2)	0.20	110 (37.9)
Left‐sided colon	44 (31.0)	55 (38.0)		99 (34.1)
Rectum	46 (32.4)	34 (23.0)		80 (27.6)
Not evaluated	0	1 (0.68)		1 (0.35)
Chemotherapy regimen, *n* (%)				
FLV	52 (36.6)	61 (41.2)	0.50	113 (39.0)
FLOX	41 (28.9)	34 (23.0)		102 (35.2)
FLIRI	49 (34.5)	53 (35.8)		75 (25.9)
Tumor response, *n* (%)				
Complete response	2 (1.4)	10 (6.8)	0.14	12 (4.1)
Partial response	37 (26.1)	34 (23.0)		71 (24.5)
Stable disease	67 (47.2)	68 (45.9)		135 (46.6)
Progressive disease	36 (25.4)	35 (23.6)		71 (24.5)
Not evaluated	0	1 (0.68)		1 (0.34)

^a^ At time of primary surgery.

^b^ At time of palliative treatment start.

### 3.1 Gene Expression in Primary Tumor Tissues

The median expression levels of each analyzed gene in primary tumors across the entire patient cohort are depicted in Figure S1. Expression of *ABCC3*, *RFC‐1*, and *PCFT*, which encode cell membrane folate transporters, was lower than expression of the mitochondrial folate transporter, *MFT*. The *MTHFD2* gene, encoding a mitochondrial enzyme that converts 5,10‐MeTHF to 10‐formyltetrahydrofolate, was highly expressed, as was *TYMS*, encoding the 5‐FU target enzyme that utilizes 5,10‐MeTHF as a cofactor.

**FIGURE 2:**
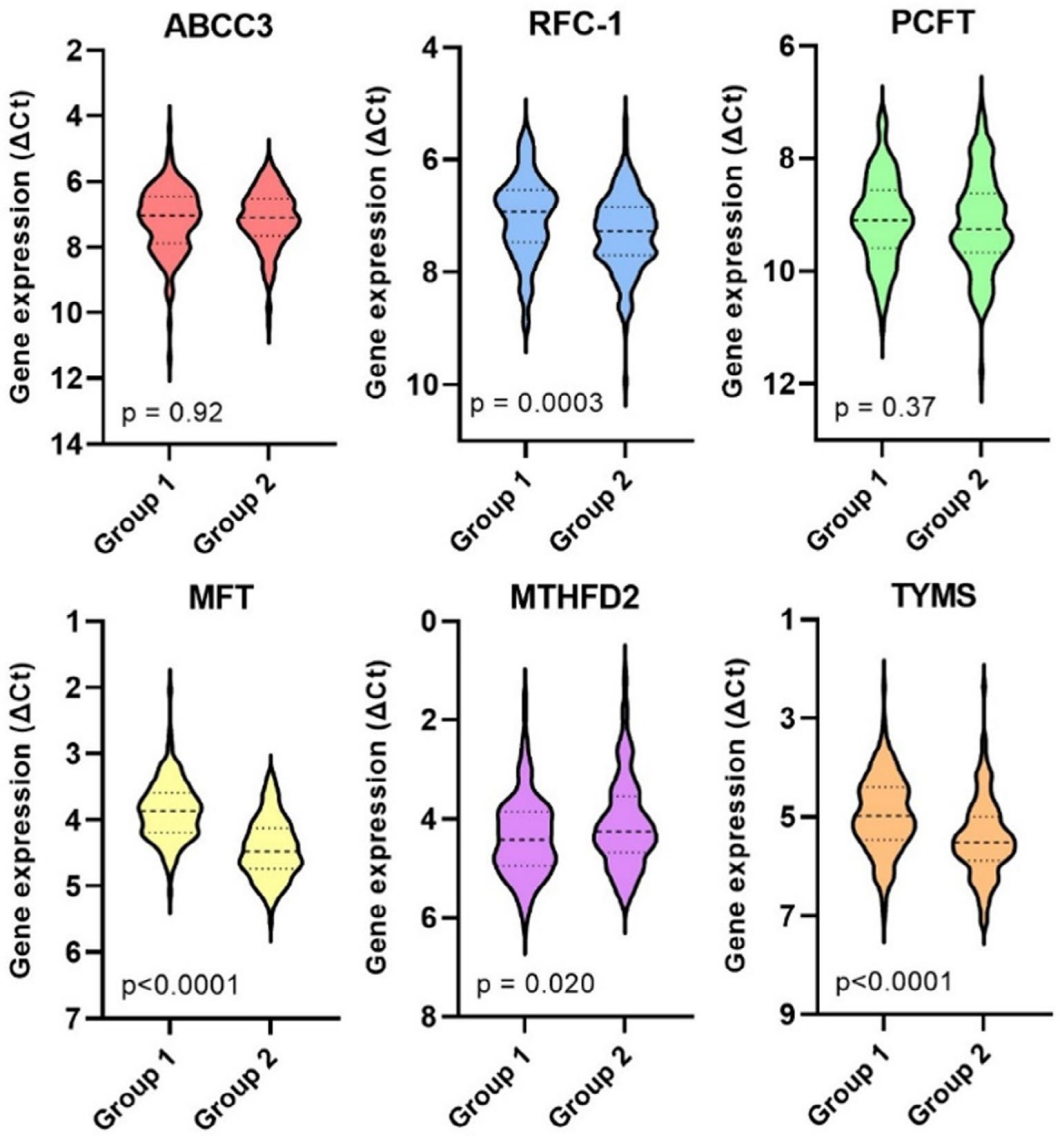
Differences in gene expression according to colorectal cancer stages at primary surgery (group 1 = stage I–III, group 2 = stage IV). Expression levels are presented as violin plots with median values and interquartile ranges depicted as horizontal lines. As shown, group 2 exhibited significantly lower expression levels of *RFC‐1*, *MFT*, and *TYMS*, alongside higher expression of *MTHFD2* compared to group 1.

As shown in Figure 2, group 2 exhibited significantly lower expression levels of *RFC‐1*, *MFT*, and *TYMS*, alongside higher expression of *MTHFD2* compared to group 1.

### 3.2 Gene Expression According to Early Versus Late Relapse in Group 1

**FIGURE 3:**
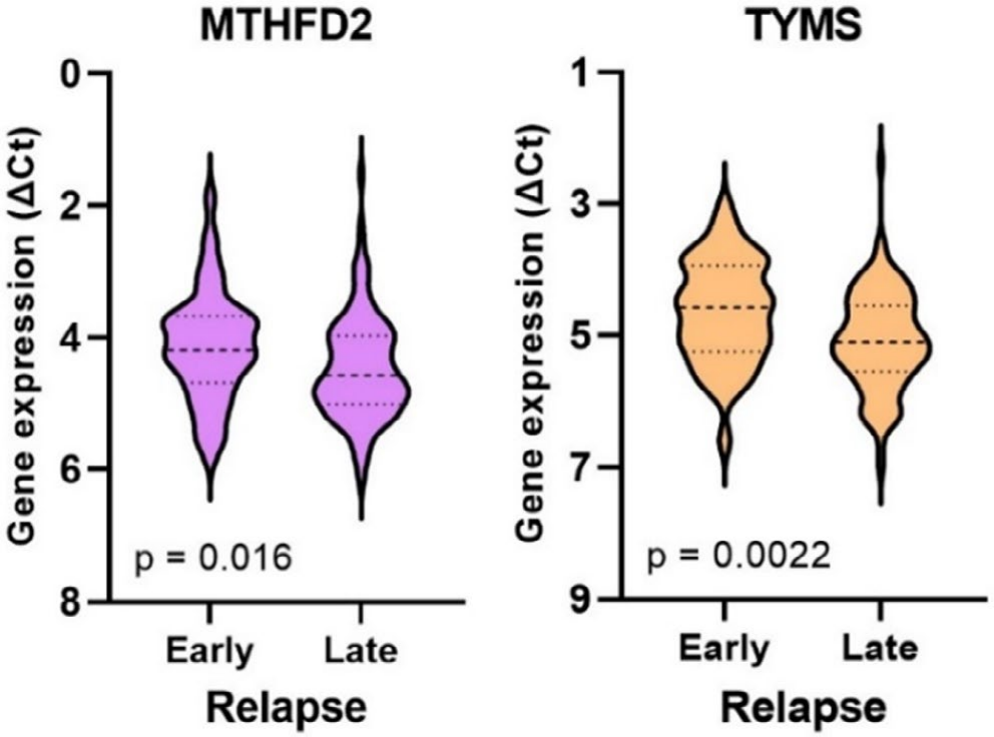
Differences in *MTHFD2* and *TYMS* gene expression based on whether patients in group 1 experienced an early or late relapse. Expression levels are presented as violin plots with median values and interquartile ranges depicted as horizontal lines. As shown, tumors from patients who experienced an early relapse exhibited significantly higher expression levels of *MTHFD2* and *TYMS* compared to those from patients with a late relapse.

Gene expression was analyzed concerning whether patients in group 1 experienced an early or late relapse (≤ 1 year vs. > 1 year after primary surgery). The analysis revealed that 41 out of 142 patients (29%) encountered an early relapse, which was significantly associated with high expression of *MTHFD2* and *TYMS* (Figure 3). Patients who experienced early relapse did not show significant differences in age, gender, tumor location, tumor differentiation, or disease stage compared to those with late relapse. However, it was noted that all patients with stage I disease had a late relapse.

### 3.3 Gene Expression Correlations

The correlation between the expression of the genes was assessed in groups 1 and 2, respectively, and the results are summarized as heatmaps in Figure S2. Only weak correlations were identified. The median percentage of tumor epithelial cells within analyzed tissue sections was 40% (range 10%–100%) and did not differ between groups 1 and 2. There were no strong correlations between the percentage of tumor epithelial cells and gene expression (Figure S2).

### 3.4 Gene Expression According to Age, Sex, and Tumor Differentiation

There was no correlation between the age of patients and the tumoral expression of any analyzed gene within the entire study cohort, nor within groups 1 or 2. Expression of *RFC‐1* was higher in tumors deriving from female compared to male patients (*p* = 0.023) and associated with patients of group 1 (*p* = 0.022). The expression of *MTHFD2* was significantly higher in poorly differentiated tumors compared to well/moderately differentiated or mucinous tumors (*p* = 0.040) and, once again, was linked to group 1 (*p* = 0.012). Furthermore, *TYMS* expression was higher in poorly differentiated tumors from patients in group 1 compared to well/moderately differentiated or mucinous tumors (*p* = 0.010). Mucinous tumors analyzed in the entire cohort exhibited a lower expression of *MFT* compared to non‐mucinous tumors (*p* = 0.0087). No significant difference was observed in the expression of the other genes regarding tumor differentiation.

### 3.5 Gene Expression According to Tumor Location

In rectal cancer compared to colon cancer, *ABCC3*, *RFC‐1*, and *MTHFD2* expression was significantly lower, while *TYMS* expression was higher. Specifically, *ABCC3* and *TYMS* expression in rectal cancer were notably different in group 1 (*p* = 0.045 and *p* = 0.0056, respectively), whereas lower *RFC‐1* expression was observed in both group 1 (*p* = 0.011) and group 2 (*p* = 0.0087). Given that rectal cancer patients often receive preoperative radiotherapy, which can impact gene expression, these patients were divided into two subgroups based on radiotherapy status (no/yes). *ABCC3* expression was significantly lower (*p* < 0.0001) and *TYMS* expression significantly higher (*p* = 0.0006) in rectal tumors subjected to radiotherapy (*n* = 43) compared to those that were not (*n* = 36, Figure S3). However, there was no difference in *RFC‐1* or *MTHFD2* expression between these subgroups. Additionally, there was no difference in the expression of any analyzed gene between rectal cancer not subjected to radiotherapy and colon cancer.

### 3.6 Association Between Gene Expression in Primary Tumors and Tumor Response

**FIGURE 4:**
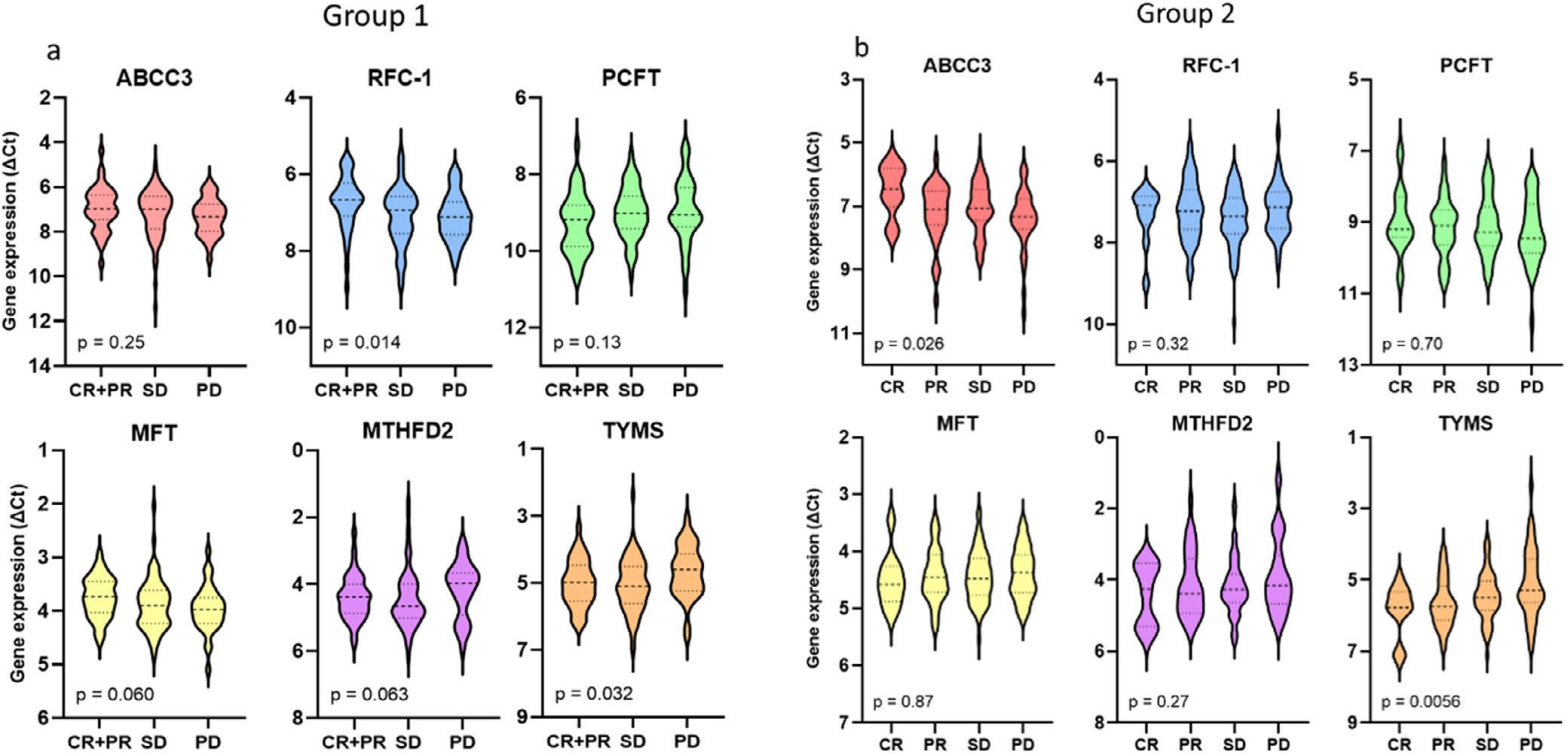
Differences in gene expression in primary tumors of (a) group 1 and (b) group 2 according to tumor response. Expression levels are presented as violin plots with median values and interquartile ranges depicted as horizontal lines. CR, complete response (*n* = 10); PR, partial response (*n* = 34); SD, stable disease (*n* = 68); PD, progressive disease (*n* = 35). As shown, a better tumor response was associated with high RFC‐1 and low TYMS expression in group 1, and with high ABCC3 and low TYMS expression in group 2.

High *ABCC3* expression and low *TYMS* expression were significantly associated with better tumor response across the entire study cohort (*p* = 0.023 and *p* = 0.0009, respectively). As shown in Table 1, tumor response did not differ significantly between groups 1 and 2. In group 1, high *RFC‐1* and low *TYMS* expression were associated with better tumor response (*p* = 0.014 and *p* = 0.032, respectively). In group 2, high *ABCC3* and low *TYMS* expression were associated with improved tumor response (*p* = 0.036 and *p* = 0.018, respectively). The expression levels of all analyzed genes in the two groups, stratified by tumor response, are presented in Figure 4. Given the potential influence of preoperative radiotherapy on *ABCC3* and *TYMS* expression, the statistical analysis was repeated after excluding rectal cancer patients who received radiotherapy. The results confirmed that the relationship between tumor response and gene expression remained statistically significant.

### 3.7 Multivariate Models

A multivariate Cox proportional hazard model was used to assess potential associations between the analyzed covariates and 3‐year PFS. A stepwise selection of variables based on AIC was then performed to identify a minimal set of variables associated with PFS. The following covariates were included in Model 1: sex (male or female), age, ECOG performance status at the start of palliative treatment (0 or 1/2), disease stage (I–IV), tumor differentiation (high/moderate, low, or mucinous), tumor location (right‐sided colon, left‐sided colon, or rectum), percentage of tumor epithelial cells in the specimen, *ABCC3*, *RFC‐1*, *PCFT*, *MFT*, *MTHFD2*, and *TYMS* gene expression, and chemotherapy regimen (FLV, FLOX, or FLIRI). Covariates that were not significant in Model 1 were removed, and the remaining variables were included in Model 2. The performance of Models 1 and 2 was compared using a deviance test, which showed similar performance. Model 2 was then retained due to its lower number of covariates.

Model 2 for the entire study cohort included the covariates ECOG performance status, tumor differentiation, *MTHFD2* expression, and chemotherapy regimen, listed in descending order of impact on the PFS hazard ratio. As expected, high ECOG performance status and poor tumor differentiation were associated with worse PFS (HR 2.2 (1.7–3.0), *p* < 0.0001, and HR 1.8 (1.4–2.4), *p* < 0.0001, respectively). *MTHFD2* expression did not reach statistical significance (HR 1.14 (0.98–1.3), *p* = 0.078). The chemotherapy regimen was significantly associated with PFS, with a notably lower risk of progression following FLOX treatment compared to FLV treatment (HR 0.70 (0.52–0.95), *p* = 0.022).

**FIGURE 5:**
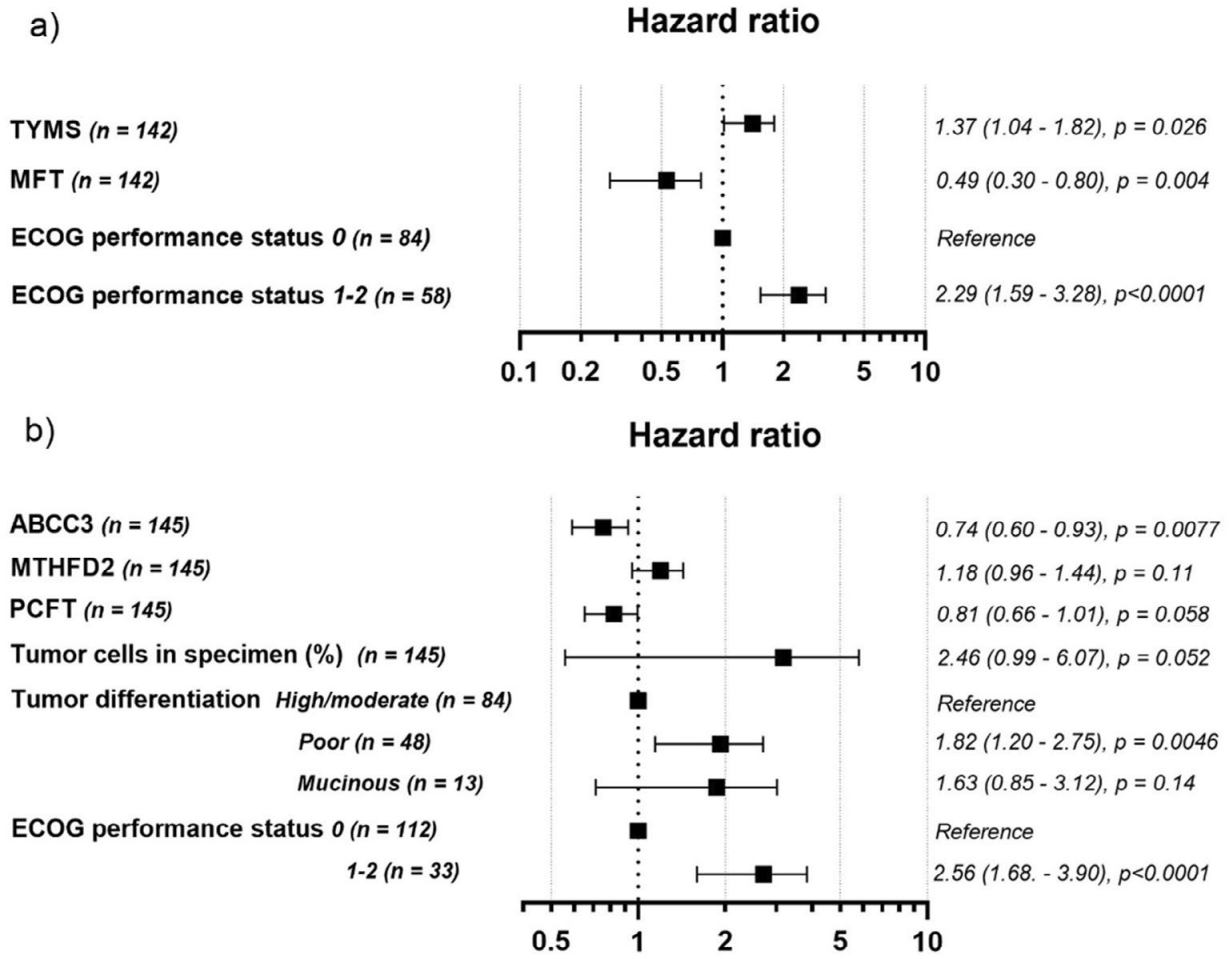
Forest plots showing hazard ratios of each covariate included in Model 2 of (a) group 1, and (b) group 2. As shown, high *MFT* and low *TYMS* expression in group 1 and high *ABCC3* expression in group 2 was significantly associated with better PFS. Tumor differentiation was a significant covariate in group 2 whereas ECOG performance status was significant in both groups.

For group 1, the model included ECOG performance status, *MFT* expression, and *TYMS* expression. For group 2, it included ECOG performance status, *ABCC3* expression, tumor differentiation, percentage of tumor epithelial cells in specimen, *PCFT* expression, and *MTHFD2* expression. The multivariate analysis indicated that high *MFT* and low *TYMS* expression in group 1 (Figure 5a), and high *ABCC3* expression in group 2 (Figure 5b), were significantly associated with better PFS. Tumor differentiation was a significant covariate in group 2, whereas ECOG performance status was significant in both groups.

**FIGURE 6:**
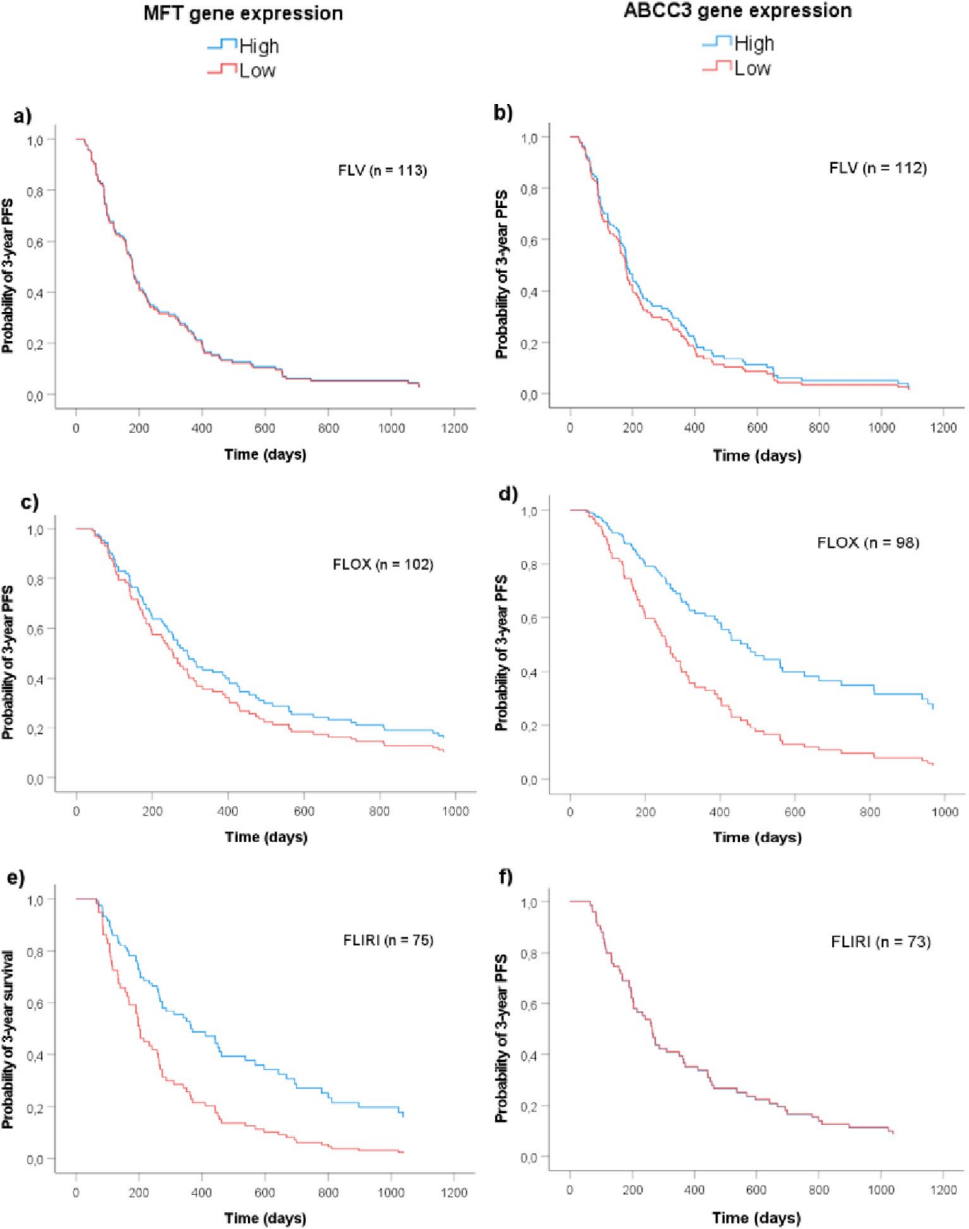
Multivariate model using covariates established in Model 2 showing the probability of PFS in patients with colorectal cancer sub‐grouped by chemotherapy and dichotomized by high and low *MFT* (a, c, e) or *ABCC3* expression (b, d, f). As depicted, high *MFT* expression was associated with better PFS of patients treated with FLIRI (*p* = 0.007) whereas high *ABCC3* expression was associated with better PFS of patients treated with FLOX (0.029).

Using covariates established in Model 2, survival functions based on maximal rank statistic estimations illustrate the association between *MFT* or *ABCC3* gene expression and PFS of patients subgrouped by treatment regimens (Figure 6). The results showed that high *MFT* expression was associated with better PFS of patients treated with FLIRI (Figure 6e, HR 0.47 (0.27–0.81), *p* = 0.007), but not of those treated with FLV or FLOX. High expression of *ABCC3*, on the other hand, was associated with better PFS of patients treated with FLOX (Figure 6d, HR 0.46 (0.23–0.92), *p* = 0.029), but not of those treated with FLV or FLIRI.

## 4 Discussion

The main purpose of the present study was to evaluate whether the expression levels of a few selected folate pathway genes in tumor tissue obtained from patients with CRC at primary surgery, that is, before the start of palliative chemotherapy, were associated with tumor response and/or 3‐year PFS. Studies were performed on the entire study cohort and separately on patients with stage I–III disease (group 1) and those with stage IV disease (group 2) at primary surgery. The hypothesis was that the significantly shorter interval from surgery (tissue sampling) to the start of chemotherapy for group 2 would result in a stronger correlation between gene expression and PFS compared to group 1. The results indicate that pretherapeutic tumoral gene expression in both groups can predict response, and possibly also outcome, of patients with mCRC subjected to the commonly used FLV‐based combination chemotherapies FLOX and FLIRI.

As expected, the ECOG performance status of patients was a strong independent prognostic factor, and as such included in Model 2 for the entire cohort as well as for group 1 and 2. The tumor differentiation grade was linked to expression of *MTHFD2* and *MFT*, with high *MTHFD2* expression in poorly differentiated tumors and low *MFT* expression in mucinous tumors. When tumor differentiation was tested as a covariate in Model 1, it was found to be significant for the entire cohort as well as group 2, but not group 1. The percentage of tumor epithelial cells in specimens correlated positively with *RFC‐1* expression and with *ABCC3* expression in group 1, indicating that these genes are either epithelial‐specific or not highly expressed in stromal cells. At least ABCC3 is normally considered to be epithelial [21]; however, stromal cells participating in tumor progression may enrich ABCC3 [22] which could explain the lack of correlation between *ABCC3* expression and percentage of tumor epithelial cells in group 2. The covariate “percentage of tumor epithelial cells” was significant for group 2 and included in Model 2.

Proximal and distal CRCs exhibit distinct gene expression profiles, which may be associated with differing biological behaviors [23]. The present study showed that the expression of *ABCC3*, *MTHFD2*, and *RFC‐1* was lower, and *TYMS* higher, in rectal compared to colon cancer. Although these differences may partly be linked to functional differences in different parts of the colorectum, there is a possibility that alterations in gene expression were induced by the preoperative radiotherapy; a lower *ABCC3* and a higher *TYMS* expression were seen in rectal tumors subjected to preoperative radiotherapy compared to radiotherapy naïve. For that reason, tumor location was tested in the statistical model but was not found to be significant.

**FIGURE 7:**
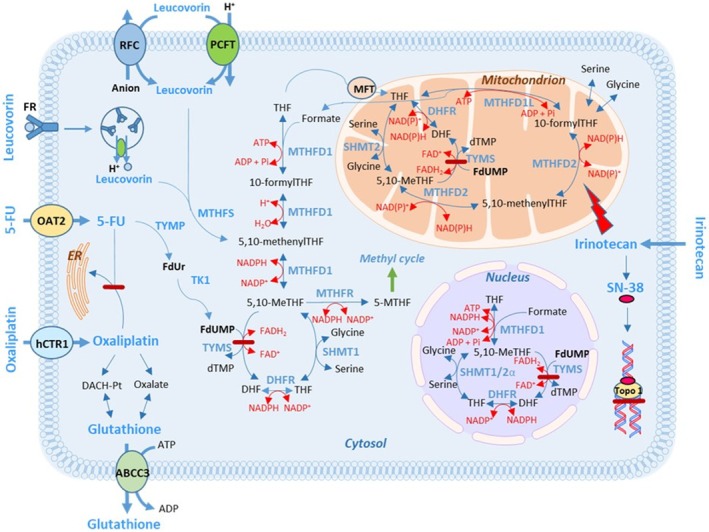
Synergistic actions between drugs and folates may be affected by the cellular redox state. Redox homeostasis is dependent on a balance between levels of oxidants and antioxidants. The latter are dependent on the generation of NADPH, which is used to maintain reduced glutathione. Abbreviations are explained in the abbreviation list.

Model 2 for the entire cohort included the covariates ECOG performance status, tumor differentiation, *MTHFD2* expression, and chemotherapy regimen. The relatively recently discovered *MTHFD2* gene encodes a mitochondrial, bifunctional enzyme which catalyzes the conversion of 5,10‐MeTHF to 10‐formylTHF in two steps [24]. The enzyme confers redox homeostasis and is thought to be responsible for mitochondrial production of both NADH and NADPH in rapidly proliferating cells (Figure 7) [25, 26]. Elevated expression of the gene has been associated with poor prognosis in solid malignancy, and it has been hypothesized that MTHFD2‐mediated NADPH homeostasis has the capacity to protect cell survival against oxidative stress, resulting in CRC progression. Interestingly, in a previous study, the highest scoring pathway frequently overexpressed in tumors was mitochondrial one‐carbon metabolism centered on MTHFD2 [27]. In the present study, *MTHFD2* expression was significant in Model 1, but there was only a trend indicating an association between high *MTHFD2* expression and worse PFS in Model 2.

In group 1, an association was seen between low *TYMS* expression and stronger tumor response, and furthermore, low *TYMS* expression was significantly associated with better PFS. Thus, the results are in line with previous studies showing that there is a significant association between the expression of the TYMS gene and the response to FLV‐based chemotherapy [28]. The 5‐FU target, TYMS, is needed for the *de novo* dTMP synthesis ensuring replication without uracil misincorporation of both mitochondrial and nuclear DNA [6]. The 5‐FU metabolite FdUMP inhibits formation of dTMP; however, overexpression of *TYMS* may indicate 5‐FU drug resistance. In addition, the association between high tumoral expression of *TYMS* and *MTHFD2* with early relapse suggests that their expression levels could serve as risk markers, supporting previous studies [24, 28]. Furthermore, the results indicate that higher tumoral expression of RFC‐1 is associated with improved tumor response to FLV‐based chemotherapy, consistent with previous reports on patients with stage III CRC [14, 15]. A high RFC‐1 expression may enhance folate uptake, leading to increased TYMS inhibition. TYMS expression was higher in Group 1 compared to Group 2, which appears contradictory to findings that associate high TYMS expression with poor prognosis. The reason for elevated TYMS expression in early versus late CRC stages is presently not known; however, this pattern aligns with RNA sequencing data from The Cancer Genome Atlas (TCGA) database, as reported by Jiang et al. [29].

The gene *MFT*, which is a member of the solute carrier 25 family of mitochondrial transporters, has previously not been studied in relation to FLV‐based treatment response. The gene encodes a protein that transfers both tetrahydrofolate (THF) and flavin adenine dinucleotide (FAD) into mitochondria, thereby regulating mitochondrial one‐carbon metabolism as well as redox balance [30]. It has been suggested that inhibition of MFT could have potential clinical applications since it could deplete levels of reduced glutathione, leading to increased oxidative stress and reduced proliferation of cancer cells [30]. In group 1, an association was seen between high *MFT* expression and better PFS. Interestingly, when testing the covariates established in model 2 in patients grouped by chemotherapy regimen and dichotomized by high/low MFT expression, high MFT expression was significantly associated with PFS only in the FLIRI group. The drug irinotecan/SN‐38 is a DNA topoisomerase I‐inhibitor (Figure 7) that also induces mitochondrial dysfunction in several ways, causing increased production of reactive oxygen species (ROS). However, many cancer types exhibit depletion of mitochondrial DNA copy number and reduced mitochondrial respiratory activity [31], probably making the cancer cells resistant to the mitochondrial effects induced by irinotecan/SN‐38 [32]. It is tempting to speculate that tumors with high *MFT* expression that responded well to FLIRI had a high number of functioning mitochondria, rendering cells more sensitive to the drug.

The ABCC3 protein belongs to the large family of ABC transporters [33]. It is regulated by the transcription factor Nrf2, which has an important role in mitochondrial function and the maintenance of cellular redox homeostasis [33, 34]. High *ABCC3* gene expression was associated with stronger tumor response in group 2 as well as a strong association with better PFS. A beneficial effect of high *ABCC3* expression was also seen in a previous study evaluating DFS in patients with stage III CRC undergoing adjuvant FLV‐based combination chemotherapy according to the Nordic bolus regime [14]. These results contradict most previous reports, which indicate that high ABCC3 expression is linked to drug resistance [35].

ABCC3 is involved in outward transport of various conjugated organic anions including drugs and toxins as well as endogenous compounds like reduced glutathione and monoglutamated folates [36]. ABCC3 might regulate reduced folate levels in tumor tissue after LV administration by extruding specific folate metabolites, and seems to have a preference for 10‐formyltetrahydrofolate [37]. This folate inhibits the enzyme methenyltetrahydrofolate synthetase (MTHFS), which is active in the first conversion step of LV to 5,10‐MeTHF. Tumors with high *ABCC3* gene expression may thus have low intracellular levels of 10‐formyltetrahydrofolate leading to a high conversion rate of LV to 5,10‐MeTHF, and a stronger inhibition of the target enzyme, TYMS. It is also possible that ABCC3 exports folates to surrounding tumor cells which could be advantageous during treatment with 5‐FU in combination with LV or other folates. It has been shown that basolateral efflux mediated by ABCC3 facilitates intestinal absorption of LV in mice [36]; however, little is known about folate transport between cells in human tissues.

Another intriguing scenario linked to a beneficial effect of high *ABCC3* expression could be the synergistic action of 5‐FU and oxaliplatin, as previously suggested by Theile et al. [38]. Based on in vitro studies, suppression of an ATPase (ATP7B) was suggested to be mediated by 5‐FU, leading to decreased sequestration of oxaliplatin into the endoplasmic reticulum (Figure 7). Furthermore, 5‐FU induces overexpression of ABCC3, thereby increasing efflux of reduced glutathione and diminishing the oxaliplatin detoxifying capacities of the cells. The oxaliplatin metabolite oxalate also decreases glutathione levels, further amplifying oxaliplatin action. This mechanism may explain why high *ABCC3* expression was associated with better PFS after FLOX‐treatment in group 2. It is not known if this mechanism is active in vivo, but high pretherapeutic *ABCC1* expression in locally advanced adenocarcinomas of the esophagus has been shown to be beneficial for treatment with platinum drugs like cisplatin [17]. *ABCC1*, like *ABCC3*, is involved in glutathione efflux [39].

Following administration of racemic LV, the intracellular folate concentrations will depend on folate availability, the efficiency of folate influx and efflux, folate polyglutamation, and intracellular folate metabolism. These processes are tightly regulated by enzymes encoded by folate‐associated genes, the expression of which is affected by alterations in the tumor environment, for example, hypoxia. It is known that reduced folates are sensitive to oxidative degradation, except for 5‐formyltetrahydrofolate, which may be a storage form of folate [40]. To prevent oxidative damage, the folates themselves [40], as well as several folate‐associated genes [26, 33, 41, 42] are redox regulators that respond to oxidative stress. Folate deficiency triggers oxidative stress and could lead to severe suppression of the mitochondrial glutathione pool [40] which may be advantageous during FLV‐based combination chemotherapy. At the same time, however, high concentrations of 5,10‐MeTHF are needed for maximal inhibition of TYMS. Modulation of the folate pool towards high levels of 5,10‐MeTHF may be accomplished by replacing LV with the novel folate arfolitixorin ([6‐R] 5,10‐MeTHF), which is given as a bolus injection in FLV‐based combination chemotherapy [1, 11].

### 4.1 Strengths and Limitations

Despite the current era of NGS, we chose to use qPCR for the gene expression analysis. qPCR is well suited for focused studies targeting specific genes of interest, as opposed to the broad approach of NGS. This targeted approach is less sensitive to DNA fragmentation and modification seen in FFPE‐fixed clinical samples and more practical and informative for hypothesis‐driven research, such as investigating the impact of folate‐associated gene expression in primary tumors on FLV‐based combination chemotherapy. Furthermore, qPCR is faster than NGS, which is particularly advantageous in clinical settings where time‐sensitive decisions are required. Additionally, qPCR provides high sensitivity and specificity for detecting low‐abundance transcripts and small changes in gene expression, which is crucial for detecting minimal residual disease in cancer. The results of the study may serve as a valuable first step in stratifying patients for the most optimal FLV‐based combination chemotherapy regimen, to avoid therapies that might not elicit a strong tumor response but could cause significant side effects. Although the study cohort was relatively large, it was still too small to evaluate tumoral gene expression after stratifying patients by both stage groups and treatment regimen. This stratification needs to be conducted in a larger patient cohort. Preoperative radiotherapy was not tested as a covariate since it only applies to rectal cancer patients and would have significantly reduced the number of analyzed patients in the models. However, the association between tumor response and *ABCC3* and *TYMS* gene expression remained significant even after excluding rectal cancer patients who underwent preoperative radiotherapy. It would be interesting, though, to investigate the potential impact of the genes in a larger cohort consisting exclusively of rectal cancer patients.

## 5 Conclusions

The results indicate that although pretherapeutic tumoral expression of specific folate‐associated genes may not serve as a universal predictive marker for FLV‐based treatment, it might predict response and possibly also outcome of patients with mCRC subjected to the FLV‐based combination chemotherapies FLOX or FLIRI. Subgrouping of patients according to early and late disease stage at diagnosis seems to be of importance since expression of *TYMS* and *MFT* was associated with PFS in group 1, whereas expression of *ABCC3* was associated with PFS in group 2. The expression levels of *MFT* and *ABCC3* were also found to be specifically associated with response to FLIRI and FLOX treatment, respectively. Differences between study cohorts regarding clinicopathological covariates and applied palliative chemotherapy regimen may lead to different conclusions when interpreting gene expression results. Folates modulate redox mechanisms and glutathione levels in tumor cells, which in turn may influence response to FLV‐based combination chemotherapy. Prospective clinical studies on patients with mCRC are warranted to validate the results of the present study.

## Author Contributions

**Elisabeth Odin:** conceptualization (equal), data curation (equal), formal analysis (equal), investigation (equal), methodology (equal), validation (equal), writing – original draft (supporting), writing – review and editing (equal). **Göran Carlsson:** conceptualization (equal), data curation (equal), formal analysis (equal), funding acquisition (supporting), investigation (equal), writing – review and editing (equal). **Pushpa Saksena:** conceptualization (equal), data curation (equal), formal analysis (equal), investigation (equal), methodology (equal), writing – review and editing (equal). **Anders Edsjö:** conceptualization (equal), data curation (equal), investigation (equal), methodology (supporting), writing – review and editing (equal). **Alessandro Di Cara:** conceptualization (equal), data curation (equal), formal analysis (equal), investigation (equal), methodology (equal), writing – review and editing (equal). **Roger Tell:** conceptualization (equal), data curation (equal), funding acquisition (supporting), methodology (supporting), project administration (supporting), validation (equal), writing – review and editing (equal). **Bengt Gustavsson:** conceptualization (equal), data curation (equal), funding acquisition (lead), resources (lead), validation (equal), writing – review and editing (equal). **Yvonne Wettergren:** conceptualization (equal), data curation (equal), formal analysis (equal), investigation (equal), methodology (equal), project administration (lead), validation (equal), visualization (lead), writing – original draft (lead), writing – review and editing (equal).

## Ethics Statement

The regional ethical review board in Gothenburg approved the study (No. 118‐15).

## Consent

Informed written and oral consent was obtained from all patients.

## Conflicts of Interest

The authors declare the following financial interests/personal relationships, which may be considered as potential competing interests: Elisabeth Odin, Göran Carlsson, Bengt Gustavsson, and Yvonne Wettergren are registered inventors of patents held by Isofol Medical AB. Elisabeth Odin and Yvonne Wettergren have received honoraria for congress participation from Isofol Medical AB. Bengt Gustavsson is the founder of Isofol Medical AB and has received honoraria for advisory board role fees and congress participation from Isofol Medical AB. Roger Tell is an employee at Isofol Medical AB. Elisabeth Odin and Bengt Gustavsson are Isofol Medical AB stockholders. Pushpa Saksena, Anders Edsjö, and Alessandro Di Cara have no financial disclosures or potential conflicts of interest to disclose.

## Supporting information


Data S1.

## Data Availability

Clinical data have been collected from patients in accordance with agreements and regulations for consent that prohibit transfer to third parties. Other datasets used or analyzed during the study are available from the corresponding author upon reasonable request.
