# Supplementary material for: Folate‐Associated Gene Expression in Primary Tumors Is Associated With Tumor Response and Progression‐Free Survival of Patients With Metastatic Colorectal Cancer Undergoing 5‐FU/Leucovorin‐Based Combination Chemotherapy
Source: Cancer Med. 2025 May 13;14(9):e70895. doi: 10.1002/cam4.70895 (PMC12070377; doi:10.1002/cam4.70895)
Supplement: Supplementary file 1 — Data S1. [file CAM4-14-e70895-s001.zip › CAM470895-sup-0001-Appendix S1.docx]

**Supplementary file 1. Extraction of RNA, preparation of cDNA, and qPCR conditions.**

**Extraction of RNA from FFPE tissue using the Qiagen AllPrep® DNA/RNA FFPE Kit (cat. no. 80234) with modifications**

Two 10 µm thick sections from the formalin-fixed paraffin-embedded tissue in the block were prepared and each section was put in a separate 1.5 ml RNase-free tube. **NOTE!** It is imperative that each of the two tubes only contains one tissue section at start of extraction and that each tube is handled separately until the last step of the proteinase K digestion (where the two supernatants are being pooled).

**Removal of paraffin**

1. Add 1 ml xylene (Fisher Scientific, article no. 11302645) to each of the two tubes containing tissue and vortex vigorously for 10 seconds.
2. Heat the tubes at 50⁰C for 3 minutes.
3. Centrifuge 2 minutes in a microcentrifuge, 16 000g.
4. Put the tubes on ice for 5 minutes.
5. Carefully remove the supernatants by pipetting without disturbing the pellets.
6. Repeat steps 1-5.
7. Add 1 ml ethanol (99.5%) to each pellet and mix for 10 seconds by vortexing.
8. Centrifuge 2 minutes, 16 000g.
9. Carefully remove the supernatants by pipetting without disturbing the pellets. Remove any residual ethanol using a fine pipet tip.
10. Keep the lids open, and leave the tubes upside down at room temperature for a minimum of 20 minutes to dry the pellets.

**Proteinase K digestion**

1. Resuspend the pellets by adding 150 μl of Buffer PKD, and flick the tubes to loosen the pellets.
2. Add 10 μl Proteinase K and mix by flicking the tubes. **Do not vortex**!
3. Incubate at 56°C for 30 minutes.
4. Put the samples on ice for 3 minutes.
5. Centrifuge for 15 minutes, 20 000g.
6. Carefully transfer the supernatant from both samples to **one** new 2 ml tube. The supernatant is used for RNA extraction. If needed, save the pellets for DNA extraction at -20°C.

**Purification of total RNA**

1. Incubate the supernatant at 80°C for 15 minutes.
2. Briefly centrifuge the tube to remove drops from the inside of the lid.
3. Add 320 μl Buffer RLT to adjust binding conditions, and mix by pipetting up and down.
4. Add 1120 μl ethanol (99.5%) and mix well by pipetting up and down several times.

The following steps of the RNA purification procedure were according to those described in the AllPrep® DNA/RNA FFPE Handbook. The concentration of the purified RNA was analyzed on a spectrophotometer (DropSense96, Trinean, or Lunatic, Unchained Labs).

**cDNA synthesis using the SuperScript^TM^ VILO^TM^ cDNA Synthesis Kit with modifications**

RNA was reverse-transcribed into cDNA using the SuperScript^TM^ VILO^TM^ cDNA Synthesis Kit (Thermo Fisher Scientific, cat. No. 11754250) in 45 µl reactions according to ***Table 1*** and ***Table 2*** below. All incubations for the cDNA synthesis were performed on T-100 cycler (BioRad). The cDNA samples were diluted 5x before running the qPCR.

| *Table 1. Temperature program for cDNA synthesis* | | | |
| --- | --- | --- | --- |
| Step | **Time** | **Temperature** | **Cycles** |
| *Activation* | 10 min | 25°C | 1 |
| *RT* | 60 min | 42°C | 1 |
| *Inactivation of enzyme* | 5 min | 85° | 1 |
|  | ∞ | 4°C (hold) |  |

| *Table 2. Mastermix protocol for VILO kit cDNA synthesis* | | | |
| --- | --- | --- | --- |
| Reagents | **Stock conc.** | **Volume (µl)** | **Final conc.** |
| *RNase-free H_2_0* | - | 2.5 | - |
| *Spike I template* | - | 4 | - |
| *VILO™ Reaction Mix* | 5X | 9 | 1X |
| *Superscript™ Enzyme Mix* | 10X | 4.5 | 1X |
| *Template RNA (≤500 ng/µl)* | - | 25 | - |
| Total reaction volume |  | **45** |  |

**qPCR analysis**

The qPCR was set up in duplicates in 384-well plates using a Nanodrop II (GC Biotech). qPCR reactions were performed using TATAA Probe® GrandMaster Mix (#TA02, TATAA Biocenter AB) and mastermixes were prepared using the setup in ***Table 3***. The qPCR was run on a QuantStudio 12 K Flex Real-Time PCR system (Thermo Fisher Scientific) according to a standard protocol using the temperature cycling program shown in ***Table 4***.

| *Table 3. qPCR mastermix protocol for one reaction* | | |
| --- | --- | --- |
| Component | **Volume (µl)** | **Stock conc.** |
| *TATAA Probe Grandmaster® Mix - Low Rox* | 2.5 | 2X |
| *Water (nuclease-free)* | 0.25 | - |
| *TaqMan assay mix* | 0.25 | 20X |
| *Template* | 2 | - |
| *Total volume* | **5** |  |

| *Table 4. Thermal protocol for qPCR* | | | | |
| --- | --- | --- | --- | --- |
| Step | **Time** | **Temperature** | **Cycles** |  |
| *Activation* | 60 s | 95°C | 1 |  |
| *Denaturation* | 5 s | 95°C | 45 |  |
| *Annealing/Extension* | 30 s | 60°C |  |  |
