# Supplementary material for: Folate‐Associated Gene Expression in Primary Tumors Is Associated With Tumor Response and Progression‐Free Survival of Patients With Metastatic Colorectal Cancer Undergoing 5‐FU/Leucovorin‐Based Combination Chemotherapy
Source: Cancer Med. 2025 May 13;14(9):e70895. doi: 10.1002/cam4.70895 (PMC12070377; doi:10.1002/cam4.70895)
Supplement: Supplementary file 1 — Data S1. [file CAM4-14-e70895-s001.zip › CAM470895-sup-0002-Appendix S2.docx]

**SUPPLEMENTARY FIGURES AND TABLES**

**Supplementary Figure S1.** Gene expression levels in colorectal tumors obtained at primary surgery. Expression levels are presented as violin plots with median values and interquartile ranges depicted as horizontal lines. The expression of *ABCC3*, *RFC-1*, and *PCFT* were lower than the expression of *MFT*, *MTHFD2*, and *TYMS*.


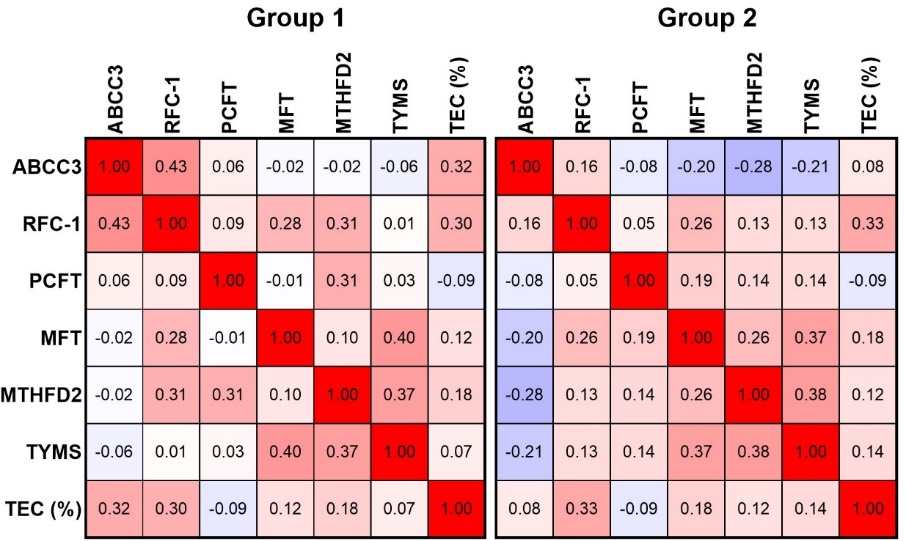


**Supplementary Figure S2**. Heat maps showing gene expression correlations in group 1 and 2, respectively, as well as correlations between gene expression and percentage of tumor epithelial cells (TEC) in specimens. Values in squares indicate the Pearson correlation coefficients (r). Positive correlation = red, negative correlation = blue.

**Supplementary Figure S3**. *ABCC3* and *TYMS* gene expression in primary tumors from rectal cancer patients by preoperative radiotherapy (no/yes). Expression levels are presented as violin plots with median values and interquartile ranges depicted as horizontal lines. As shown, the *ABCC3* expression was significantly lower, whereas *TYMS* expression was higher, in rectal tumors subjected to preoperative radiotherapy.

| Supplementary Table 1. List and assay ID of analyzed genes | | | |
| --- | --- | --- | --- |
| Gene category | Gene | Gene name | Assay ID |
| Folate transport | *ABCC3/MRP3* | ATP-binding cassette, subfamily C (CFTR/MRP), member 3 | Hs00358656_m1 |
|  | *SLC19A1/RFC-1* | Solute carrier family 19 (folate transporter), member 1/Reduced folate carrier 1 | Hs00953344_m1 |
|  | *SLC46A1/PCFT* | Solute carrier family 46 (folate transporter), member 1/Proton coupled folate transporter | Hs00611081_m1 |
|  | *SLC25A32/MFT* | Solute carrier family 25, member 32/Mitochondrial folate transporter | Hs00229219_m1 |
|  |  |  |  |
| Folate metabolism | *MTHFD2* | Methylenetetrahydrofolate dehydrogenase (NAPD+ dependent) 2 | Hs00759197_s1 |
| 5-FU target | *TYMS* | Thymidylate synthase | Hs00426586_m1 |
| House-keeping | *ACTB* | β-actin | Hs01060665_g1 |
|  | *PPIA* | Peptidylpropyl isomerase A | Hs99999904_m1 |
| ATP, adenosine triphosphate; CFTR, cystic fibrosis transmembrane conductance regulator; MRP, multidrug resistance protein; NADP, nicotinamide adenine dinucleotide phosphate | | | |
